# Knowledge, Attitude, and Practice of Saudi Medical, Nursing, and Pharmacy Students and Interns Regarding Antidepressant Drugs and Drug-Induced Serotonin Syndrome

**DOI:** 10.7759/cureus.51335

**Published:** 2023-12-30

**Authors:** Sahar M Elashmony, Bashar W Sheikh, Rafal A Brashi, Ziyad Almalki, Atheer Alharthi, Shaima Alghuraybi, Saja Bakhshwaen, Mohammad H Alsharif

**Affiliations:** 1 Pharmacology and Toxicology, Umm Al-Qura University, College of Medicine, Al-Qunfudah, SAU; 2 Medical Pharmacology Department, Cairo University, College of Medicine, Cairo, EGY; 3 College of Medicine, Umm Al-Qura University, Makkah, SAU; 4 College of Pharmacy, Umm Al-Qura University, Makkah, SAU; 5 College of Medicine, King Abdulaziz University, Faculty of Medicine, Rabigh, SAU; 6 Preventive Medicine, King Khalid University Hospital, Riyadh, SAU; 7 College of Medicine and Surgery, Batterjee Medical College, Jeddah, SAU

**Keywords:** serotonergic medications, psychopharmacology, side effects, mental health, medical education, medical students

## Abstract

Background and aim

Antidepressant drugs are commonly used to treat depressive disorders and anxiety. However, they can cause side effects, including drug-induced serotonin syndrome, which is a potentially life-threatening condition. It is essential to understand the level of knowledge of healthcare professionals who are likely to prescribe and administer these medications. This article aims to assess the knowledge of Saudi medical, nursing, and pharmacy students and interns regarding antidepressant drugs and drug-induced serotonin syndrome.

Methods

A cross-sectional survey was conducted among medical, nursing, and pharmacy students and interns in Saudi Arabia. A self-administered questionnaire was used to collect data from participants. The questionnaire consisted of three sections: demographic information, knowledge about antidepressants, and knowledge about serotonin syndrome.

Results

A total of 425 participants were included in the study. The median knowledge score for antidepressants and serotonin syndrome was moderate to good, with median scores of 18 out of 23 (IQR: 16-20) and eight out of 12 (IQR: 6-10), respectively. However, more than half of the participants had sufficient knowledge about these topics, with only 227 (53.4%) and 264 (62.1%) having sufficient knowledge about antidepressants and serotonin syndrome, respectively. Regarding serotonin syndrome, males had a significantly higher proportion of sufficient knowledge compared to females, 86 (70.5%) out of 122 vs. 178 (58.7%) out of 303 (p=0.024), respectively. Medical students/interns had a significantly higher proportion of sufficient knowledge about antidepressants compared to nursing students/interns. According to the academic year, interns had the highest proportion of sufficient knowledge.

Conclusion

The current study revealed that Saudi medical, nursing, and pharmacy students and interns had moderate to good levels of knowledge about antidepressants and serotonin syndrome. The participating students had slightly better knowledge about serotonin syndrome in comparison to knowledge about antidepressants. Further research is needed to identify the causes of the knowledge gap and develop targeted interventions to address these causes. Educational efforts to ensure the safe and effective use of antidepressants are needed.

## Introduction

Antidepressants like selective serotonin reuptake inhibitors (SSRIs) treat depressive disorders and anxiety by increasing neurotransmitter concentration in the synaptic cleft. Psychiatrists use SSRIs as first-line antidepressants due to their effectiveness, tolerability, and safety in high doses. SSRIs are also used to treat other psychiatric disorders. Commonly used SSRIs are citalopram, escitalopram, fluvoxamine, fluoxetine, sertraline, and paroxetine [1]. Antidepressants can cause side effects, such as gastrointestinal symptoms, somatic symptoms, and hormonal imbalances. Gastrointestinal symptoms include indigestion, nausea, diarrhea, and constipation. Somatic symptoms can include vertigo, low blood pressure, headache, and blurred vision, while hormonal imbalances may cause excessive sweating, heat stroke, and dry mouth [2].

Serotonin syndrome is a potentially life-threatening condition caused by excessive serotonergic activity in the central nervous system due to medication use. It is characterized by a range of symptoms, which can include changes in mental status, autonomic hyperactivity, and neuromuscular abnormalities [3]. Serotonin syndrome is diagnosed based on clinical findings, and there is no single test to confirm the diagnosis. Treatment involves discontinuation of the serotonergic medication and supportive care. Severe cases of the syndrome can lead to complications such as seizures, rhabdomyolysis, myoglobinuria, metabolic acidosis, and renal failure [3]. Mild cases resolve within 24 to 72 hours of conservative care and withdrawing serotonergic medications, while moderate to severe cases require hospitalization, depending on the symptoms [4].

This study aims to fill a gap in the existing literature by assessing the knowledge, attitude, and practices of Saudi medical, nursing, and pharmacy students and interns regarding antidepressant drugs and drug-induced serotonin syndrome. To the best of our knowledge, no previous studies have been conducted on this topic. Given the prevalence of mental health disorders and the widespread use of antidepressants, it is essential to understand the level of knowledge and awareness among future healthcare professionals who are likely to prescribe and administer these medications.

## Materials and methods

Study design and population

This cross-sectional study utilized a structured online questionnaire for data collection.

The study population consists of medical, nursing, and pharmacy students and interns in Saudi Arabia. Inclusion criteria were medical, nursing, pharmacy students, interns, and pharmacists in Saudi Arabia. Exclusion criteria were students or interns who were unwilling to participate in the study.

Sample size calculation was based on the total number of medical, nursing, and pharmacy students and interns in the study area. The sample size was calculated using the formula (Z^2^pq)/d^2^, where Z=1.96 (for a 95% confidence interval), p=0.5 (the proportion in the population estimated to have the outcome of interest), q=1-p, and d=0.05 (the margin of error). The estimated sample size was 384 participants. To account for potential non-response, a total of 425 participants were recruited. Convenience sampling was used to recruit participants to reach the desired sample size. Participants were recruited from various universities and healthcare institutions in Saudi Arabia.

Data collection

A self-administered questionnaire was used to collect data from participants. The questionnaire consisted of three sections: demographic information, knowledge about antidepressants, and knowledge about serotonin syndrome. The knowledge sections included a series of multiple-choice questions. The researchers developed the questionnaire based on a review of the literature and expert opinions. The questionnaire underwent face validation by two experts in the field of psychiatry, resulting in confirmation of the validity of the employed items.

Data collection was conducted between June 2023 and August 2023. The questionnaires were distributed to participants electronically through Google Forms (Google, Inc., Mountain View, CA) and shared through social media. Participants were informed that their participation was voluntary and confidential.

Statistical analysis

SPSS version 20.0 (IBM Corp, Armonk, NY) was used to perform the analyses. Descriptive statistics were used to summarize the demographic characteristics of the sample. The knowledge scores were calculated for each participant based on the number of correct answers. The total knowledge scores were categorized into a binary variable (sufficient vs. insufficient) based on the median. The cutoff thresholds were established at medians of 18 out of 23 for questions about knowledge of antidepressants and eight out of 12 for questions related to serotonin syndrome knowledge. The chi-square test was used to compare percentages of categorical variables within each group. However, Fisher's exact test was used for variables with less than five observations in any category. A p-value less than 0.05 was considered statistically significant.

The Chronbach alpha coefficient was calculated to test the reliability of the knowledge items. Normality assumption was checked for the sum of knowledge about serotonin and antidepressants using the Shapiro-Wilk test. However, the sum of knowledge scores did not meet the normality assumptions.

Ethical considerations

The Institutional Review Board (IRB) of Umm Al-Qura University approved the study on June 22, 2023, with approval no. HAPO-02-K-012-2023-06-1681. Before the completion of the questionnaire, informed consent was obtained from all participants. Participant confidentiality was ensured by using identification codes rather than personal names. The collected data were utilized solely for research purposes only.

## Results

A total of 425 responses were analyzed. The majority of participants were Saudi Arabians, 412 (96.9%), and females, 303 (71.3%). Most participants were either medical students or interns, 357 (84.0%), while the remaining participants were nursing students or interns, pharmacy students or interns, pharmacists, or other occupations. About one-third of the participants, 135 (31.8%), were fifth-year students, and 262 (61.8%) attended Umm Al-Qura University. The sociodemographic characteristics of the sample are presented in Table 1).

**Table 1:** Sociodemographic characteristics of the participants

Variables	Groups	n (%)
Gender	Female	303 (71.3%)
	Male	122 (28.7%)
Nationality	Saudi	412 (96.9%)
	Non-Saudi	13 (3.1%)
Occupation	Medical student/ intern	357 (84.0%)
	Nursing student/ intern	33 (7.8%)
	Pharmacy student/ intern	28 (6.6%)
	Pharmacist	3 (0.7%)
	Other	3 (0.7%)
Academic year	1st year	3 (0.7%)
	2nd year	51 (12.0%)
	3rd year	51 (12.0%)
	4th year	80 (18.8%)
	5th year	135 (31.8%)
	6th year	44 (10.4%)
	7th year (intern)	61 (14.4%)
University where they study	Umm Al-Qura University	262 (61.8%)
	King Abdulaziz University	76 (17.9%)
	University of Jeddah	30 (7.1%)
	King Saud University	12 (2.8%)
	Qassim University	14 (3.3%)
	Taibah University	12 (2.8%)
	Other	18 (4.2%)

**Figure 1:**
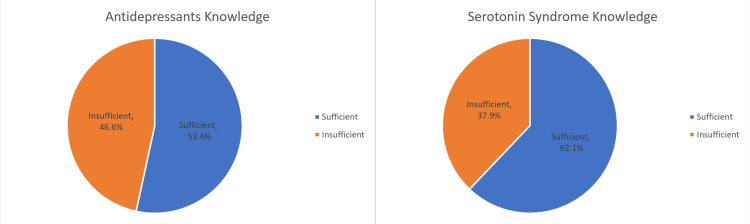
Level of knowledge among the participants regarding antidepressants and serotonin syndrome

The median score for knowledge about antidepressants was 18 out of 23 (IQR: 16-20), whereas the median score for knowledge about serotonin syndrome was eight out of 12 (IQR: 6-10). More than half of the participants had sufficient knowledge about serotonin syndrome, 264 (62.1%), and antidepressants, 227 (53.4%). Knowledge levels are presented in Figure 1.

The total scores for knowledge of antidepressants and serotonin syndrome were assessed for reliability using the Cronbach alpha coefficient. The reliability coefficient for the antidepressant knowledge score was 0.85, while the serotonin syndrome knowledge score had a reliability coefficient of 0.69.

The study employed two knowledge scales: one about antidepressant medications in general, including mechanism of action and side effects, and the other scale focused on serotonin syndrome. The accurate responses to questions about serotonin syndrome and antidepressant medications are shown in Tables 2 and 3.

**Table 2:** True/false statements used to measure the participants' knowledge of serotonin syndrome T - true; F - false

Statements	True/False	Correct answers, n (%)
Serotonin syndrome can be caused by illicit drugs such as ecstasy and amphetamines	T	175 (41.2%)
Serotonin syndrome is life-threatening	T	234 (55.1%)
Serotonin syndrome is a medical emergency	T	245 (57.6%)
There is a certain test for diagnosing serotonin syndrome	F	68 (16%)
The symptoms of serotonin syndrome can range from mild to severe (life-threatening)	T	265 (62.4%)
Serotonin syndrome is managed mainly by supportive care through antihypertensive medications, fluids, and benzodiazepines	T	171 (40.2%)
Genetics can make certain patients at risk of serotonin syndrome	T	195 (45.9%)
Serotonin syndrome can result from only antidepressant medication combinations such as more than one SSRI drug or SSRIs and SNRIs combination	T	103 (24.2%)
Serotonin syndrome can result from antidepressant medication combinations with other medications, such as ciprofloxacin or fluconazole	T	127 (29.9%)
Serotonin syndrome can result from the consumption of grapefruit with certain antidepressant medications	T	80 (18.8%)
A washout period is a method of serotonin syndrome prevention	T	143 (33.6%)

**Table 3:** Questions used to measure the participants' knowledge of antidepressants SSRIs - selective serotonin reuptake inhibitors; SNRIs - serotonin and norepinephrine reuptake inhibitors; TCAs - tricyclic antidepressants; MAOIs - monoamine oxidase inhibitors; MAO - monoamine oxidase; NE - norepinephrine; 5-HT - 5-hydroxytryptamine (serotonin); T - true; F - false

Questions		True/False	Correct answers, n (%)
Which of the following best describes the mechanism of SSRIs?	a) Block serotonin reuptake only	T	231 (54.4%)
	b) Block serotonin and norepinephrine reuptake	F	
	c) Block norepinephrine reuptake only	F	
	d) Irreversible inhibitory effect of MAO and increased duration of NE, dopamine, and 5HT to remain in the synaptic cleft by preventing their degradation	F	
Which of the following best describes the mechanism of SNRIs	a) Block serotonin reuptake only	F	175 (41.2%)
	b) Block serotonin and norepinephrine reuptake	T	
	c) Block norepinephrine reuptake only	F	
	d) Irreversible inhibitory effect of MAO and increased duration of NE, dopamine, and 5HT to remain in the synaptic cleft by preventing their degradation remain in the synaptic cleft by preventing their degradation	F	
Which of the following best describes the mechanism of TCAs	a) Block serotonin reuptake only	F	61 (14.4%)
	b) Block serotonin and norepinephrine reuptake	F	
	c) Block norepinephrine reuptake only	T	
	d) Irreversible inhibitory effect of MAO and increased duration of NE, dopamine, and 5HT to remain in the synaptic cleft by preventing their degradation	F	
Which of the following best describes the mechanism of MAOIs	a) Block serotonin reuptake only	F	181 (42.6%)
	b) Block serotonin and norepinephrine reuptake	F	
	c) Block norepinephrine reuptake only	F	
	d) Irreversible inhibitory effect of MAO and increased duration of NE, dopamine, and 5HT to remain in the synaptic cleft by preventing their degradation	T	
Some antidepressants are indicated for conditions other than depression		T	292 (68.7%)
Amitriptyline is an antidepressant drug		T	159 (37.4%)
Methotrexate is an antidepressant drug		F	125 (29.4%)
Bupropion is an antidepressant drug which is used as an aid long-term for smoking cessation		T	129 (30.4%)
Fluoxetine is an antidepressant drug		T	192 (45.2%)
Carbamazepine is an antidepressant drug		F	135 (31.8%)
Intranasal esketamine (Spravato®) works on non-competitive N-methyl-D-aspartate (NMDA) glutamate receptors in the brain		T	54 (12.7%)
Intranasal esketamine (Spravato®) is approved for adults with treatment-resistant depression (TRD) who have been through multiple treatment cycles without relief		T	52 (12.2%)
The effect of intranasal ketamine is produced within hours of administration of intranasal esketamine (Spravato®)		T	55 (12.9%)
The expected time to reach peak plasma concentration is 20 to 40 minutes of intranasal esketamine (Spravato®)		T	53 (12.5%)
Esketamine nasal spray is used along with other oral antidepressants		T	50 (11.8%)
Contraindication of esketamine	a) Vascular disease or arteriovenous malformation	T	207 (58.6%)
	b) Intracerebral hemorrhage	T	
	c) Hypertension	T	
	d) Pulmonary conditions	T	
	e) Pregnancy and lactation	F	

Regarding serotonin syndrome, males had a significantly higher proportion of sufficient knowledge compared to females, 86 (70.5%) out of 122 vs. 178 (58.7%) out of 303 (p=0.024), respectively.

A significant association was found between college and level of knowledge about serotonin syndrome (p=0.009). Pharmacy students had the highest proportion of sufficient knowledge, 25 (89.3%). Knowledge levels also significantly differed based on the academic year, with seventh-year (intern) students having the highest proportion of sufficient knowledge regarding serotonin syndrome, 47 (77%, p=0.023). Detailed results are shown in Table 4.

**Table 4:** Crosstabulation of sociodemographic factors with the levels of knowledge regarding serotonin syndrome * statistically significant

Sociodemographic factors		A sum of knowledge about serotonin syndrome		
		Sufficient n (%)	Insufficient n (%)	p-value
Gender	Female	178 (58.7%)	125 (41.3%)	0.024*
	Male	86 (70.5%)	36 (29.5%)	
Nationality	Saudi	257 (62.4%)	155 (37.6%)	0.532
	Non-Saudi	7 (53.8%)	6 (46.2%)	
Occupation	Medical student/ intern	218 (61.1%)	139 (38.9%)	0.009*
	Nursing student/ intern	17 (51.5%)	16 (48.5%)	
	Pharmacy student/ intern	25 (89.3%)	3 (10.7%)	
	Pharmacist	2 (66.7%)	1 (33.3%)	
	Other	2 (50.0%)	2 (50.0%)	
Academic year	1st year	2 (66.7%)	1 (33.3%)	0.023*
	2nd year	28 (54.9%)	23 (45.1%)	
	3rd year	29 (56.9%)	22 (43.1%)	
	4th year	42 (52.5%)	38 (47.5%)	
	5th year	83 (61.5%)	52 (38.5%)	
	6th year	33 (75.0%)	11 (25.0%)	
	7th year (intern)	47 (77.0%)	14 (23.0%)	
University where they study	Umm Al-Qura University	156 (59.5%)	106 (40.5%)	0.033*
	King Abdulaziz University	41 (53.9%)	35 (46.1%)	
	University of Jeddah	22 (73.3%)	8 (26.7%)	
	King Saud University	7 (58.3%)	5 (41.7%)	
	Qassim University	12 (85.7%)	2 (14.3%)	
	Taibah University	10 (83.3%)	2 (16.7%)	
	Other	3 (16.7%)	15 (83.3%)	

The results indicate that females had a significantly lower proportion of sufficient knowledge about antidepressants compared to males, 149 (49.2%) out of 303 vs. 78 (63.9%) out of 122 (p=0.024), respectively. Additionally, medical students/interns had a significantly higher proportion of sufficient knowledge when compared to nursing students/interns, 189 (52.9%) out of 357 vs. 10 (30.3%) out of 33 (p=0.009), respectively. Based on the academic year, significant differences in knowledge levels were found, with interns having the highest proportion of sufficient knowledge, 52 (85.2%; p<0.001). The study found that more than half of the participants, 227 (53.4%), had sufficient knowledge about antidepressants. Detailed results are shown in Table 5.

**Table 5:** Crosstabulation of sociodemographic factors with the levels of knowledge regarding antidepressants * statistically significant

Sociodemographic factors		A sum of knowledge about antidepressants		
		Sufficient n (%)	Insufficient n (%)	p-value
Gender	Female	149 (49.2%)	154 (50.8%)	0.006*
	Male	78 (63.9%)	44 (36.1%)	
Nationality	Saudi	222 (53.9%)	190 (46.1%)	0.272
	Non-Saudi	5 (38.5%)	8 (61.5%)	
Occupation	Medical student/ intern	189 (52.9%)	168 (47.1%)	0.009*
	Nursing student/ intern	10 (30.3%)	23 (69.7%)	
	Pharmacy student/ intern	24 (85.7%)	4 (14.3%)	
	Pharmacist	2 (66.7%)	1 (33.3%)	
	Other	2 (50.0%)	2 (50.0%)	
Academic year	1st year	2 (66.7%)	1 (33.3%)	<0.001*
	2nd year	14 (27.5%)	37 (72.5%)	
	3rd year	26 (51.0%)	25 (49.0%)	
	4th year	36 (45.0%)	44 (55.0%)	
	5th year	72 (53.3%)	63 (46.7%)	
	6th year	25 (56.8%)	19 (43.2%)	
	7th year (intern)	52 (85.2%)	9 (14.8%)	
University where they study	Umm Al-Qura University	131 (50.0%)	131 (50.0%)	<0.001*
	King Abdulaziz University	39 (51.3%)	37 (48.7%)	
	University of Jeddah	5 (41.7%)	7 (58.3%)	
	King Saud University	16 (53.3%)	14 (46.7%)	
	Qassim University	10 (71.4%)	4 (28.6%)	
	Taibah University	10 (83.3%)	2 (16.7%)	
	Other	15 (83.3%)	3 (16.7%)	

## Discussion

Serotonin syndrome incidence is reported to be between 0.6 and 2.6 cases per 10,000 persons per year [5]. In this regard, the cross-sectional study of knowledge can elaborate on practices and knowledge that can help guide policy decisions. Pouring evidence suggests that knowledge is suitable for different disease conditions, such as rehabilitation education of patients suffering from intervertebral disc herniation [6], precautionary measures to control COVID-19 [7], and incidence of hepatitis B in healthy populations [8]. Similarly, several studies have assessed knowledge, attitude, and practice in various conditions among the Saudi population [9-11].

Our study is the first to shed light on the serotonin syndrome and knowledge among medical, nursing, and pharmacy students. We found that more than half of the students had sufficient knowledge about antidepressants and serotonin syndrome. Considering the students' knowledge base, our study results are similar to another knowledge study about serotonin syndrome conducted among neuro-physicians in India. This study also found that only 46 out of 150 (31%) neuro-physicians could correctly identify the criteria of serotonin syndrome [12]. Concurrent with our study findings, another knowledge study on Saudi medical students found that only 343 out of 778 (44.1%) had sufficient mental illness knowledge. However, their attitude toward mentally ill patients was sympathetic [13]. Our findings were also consistent with a study conducted by Ahmed et al. among primary care physicians. This study found that only 200 out of 455 (44%) of the physicians had adequate knowledge about mental, neurological, and substance use; however, 425 out of 455 (93.4%) of physicians showed a positive attitude towards the patients [14].

Several studies have defined knowledge regarding depression or serotonin syndrome with the number of male or female participants, although differences between males and females were not discussed [12,14,15]. However, our study was able to reveal the differences between male and female participants in terms of sufficient knowledge about serotonin syndrome, 86 (70.5%) out of 122 vs. 178 (58.7%) out of 303 (p=0.024), respectively. In contrast, a knowledge study on COVID-19 in the Saudi population found that knowledge statements were more accurate in females (p=0.005) [16]. This may be due to the fact that knowledge, practices, and attitudes were better in females related to COVID-19 [17,18].

Our study was also able to differentiate between the level of education, i.e., medical, nursing, or pharmacy students, and knowledge levels based on the academic year of the internship. Similar to our findings, Giovani et al. also showed that knowledge of COVID-19 among Indonesian medical students was dependent on the year of study between third vs. first-year medical students and gender [19]. Our study was also able to decipher that the knowledge base about serotonin syndrome was highest among pharmacy students, followed by medical and, finally, nursing students. Similar to our findings, a study about self-medication found that pharmacy students had more knowledge base about drug information as compared to medical students (p<0.001) [20]. On the other hand, another study also presented similar results, finding that knowledge of nosocomial infections was better in medical students than in nursing students [21].

Our study of knowledge on serotonin syndrome has provided a holistic view of participants' knowledge, attitudes, and practices related to the serotonin syndrome. Thus, this allows for a comprehensive understanding of the differences between the three groups. In addition, our study also provides variations in knowledge, attitudes, and practices between medical, pharmacy, and nursing students. As the students belonged to different institutions across Saudi Arabia containing diverse groups, valuable insights could be obtained, and areas of improvement in each student group could be identified. Our study is a cross-sectional survey-based study with several limitations, such as self-reporting bias, which can limit the effectiveness of the results. Also, the study was conducted for a short period of time; thus, longitudinal data giving more information on how educational interventions helped fill the gap is missing.

Additionally, the curricula of pharmacy, medical, and nursing students are different, and they have different exposure to clinical settings. This can also introduce confounding factors and limit the ability to attribute differences. Nonetheless, ours is the first study in Saudi Arabia that compares medical, nursing, and pharmacy students' knowledge, attitude, and practice for serotonin syndrome. The valuable insights obtained can direct future targeted studies.

## Conclusions

The current study revealed that Saudi medical, nursing, and pharmacy students and interns had moderate to good levels of knowledge about antidepressants and serotonin syndrome. The participating students had slightly better knowledge of serotonin syndrome in comparison to knowledge of antidepressants. Further research is needed to identify the causes of the knowledge gap and develop targeted interventions to address these causes. Educational efforts to ensure the safe and effective use of antidepressants are needed.
